# Ticagrelor and prasugrel are independent predictors of improved long‐term survival in ACS patients

**DOI:** 10.1111/eci.13304

**Published:** 2020-09-09

**Authors:** Gloria M. Gager, Bernd Jilma, Max‐Paul Winter, Christian Hengstenberg, Irene M. Lang, Aurel Toma, Florian Prüller, Markus Wallner, Ewald Kolesnik, Dirk von Lewinski, Jolanta M. Siller‐Matula

**Affiliations:** ^1^ Department of Clinical Pharmacology Medical University of Vienna Vienna Austria; ^2^ Department of Internal Medicine II Division of Cardiology Medical University of Vienna Vienna Austria; ^3^ Clinical Institute of Medical and Chemical Laboratory Diagnostics Medical University of Graz Graz Austria; ^4^ Department of Cardiology Medical University of Graz Graz Austria; ^5^ Cardiovascular Research Center Lewis Katz School of Medicine Temple University Philadelphia PA USA; ^6^ Center for Biomarker Research in Medicine CBmed GmbH Graz Austria; ^7^ Department of Experimental and Clinical Pharmacology Centre for Preclinical Research and Technology (CEPT) Medical University of Warsaw Warsaw Poland

**Keywords:** clopidogrel, MEA, mortality, platelets, prasugrel, ticagrelor

## Abstract

**Aim:**

To investigate the long‐term clinical benefit of dual antiplatelet therapy with potent P2Y12 inhibitors compared to clopidogrel in patients with acute coronary syndrome (ACS).

**Methods:**

In this prospective multicenter observational study, we enrolled 708 patients with ACS treated with clopidogrel (n = 137), ticagrelor (n = 260) or prasugrel (n = 311). Major adverse cardiac events (MACE; over 1 year) and long‐term mortality (median: 5.6 years; interquartile range [IQR] 4.9‐6.5 years) were assessed. Multiple electrode aggregometry (MEA) was used to measure adenosine diphosphate (ADP)‐ and arachidonic acid (AA)‐induced platelet aggregation.

**Results:**

Type of P2Y12 inhibitor emerged as an independent predictor of long‐term mortality and MACE: patients treated with potent platelet inhibitors prasugrel or ticagrelor were at lower risk for long‐term mortality (adjusted hazard ratio [HR] = 0.44; 95% CI: 0.22‐0.92; *P* = .028) or MACE (adjusted HR = 0.38; 95% CI: 0.20‐0.73; *P* = .004) than those treated with clopidogrel independent from clinical risk factors. In contrast, the efficacy of clopidogrel decreased with increasing severity of ACS: platelet aggregation was 37% higher in patients with ST segment elevation myocardial infarction (STEMI) and 25% higher in patients with non‐ST elevation myocardial infarction (non‐STEMI) compared to patients with unstable angina (*P* = .039). Patients with diabetes achieved less potent ADP‐ and AA‐induced platelet inhibition under clopidogrel, compared to patients without diabetes (*P* = .045; *P* = .030, respectively).

**Conclusion:**

In the setting of ACS, treatment with ticagrelor or prasugrel reduced long‐term mortality and 1‐year MACE as compared to clopidogrel.

## 1 INTRODUCTION

Dual antiplatelet therapy (DAPT), where P2Y12 inhibitors are combined with aspirin, is the gold standard therapy in patients presenting with acute coronary syndrome (ACS).^1^ Superiority regarding ischemic outcomes of the potent P2Y12 inhibitors ticagrelor and prasugrel compared to clopidogrel is well‐known^2^ due to more consistent and faster platelet inhibition.^3^, ^4^, ^5^, ^6^, ^7^ Of note, these benefits in ischemic outcomes naturally bring an increasing risk for bleeding events.^8^, ^9^ Nevertheless, mortality rates are reduced under ticagrelor and prasugrel treatment.^10^, ^11^ Therefore, current guidelines of the European Society of Cardiology recommend potent P2Y12 inhibitors as the first‐line therapy in patients with ACS leaving clopidogrel merely for patients with contraindications to novel P2Y12 inhibitors.^12^, ^13^, ^14^, ^15^ Till date, data investigating long‐term mortality between clopidogrel, ticagrelor and prasugrel are sparse. Clinical trials that reported data so far had maximal observation durations of 30 months.^16^ In the present study, we aimed to compare long‐term mortality rates in patients receiving either clopidogrel, ticagrelor or prasugrel in the setting of ACS. Furthermore, we characterize predictors for major adverse cardiovascular events (MACE) and platelet aggregation patterns according to the P2Y12 inhibitor type.

## 2 METHODS

### 2.1 Study design

Patients with ACS from two cohort studies investigating platelet reactivity and clinical outcome were included into this analysis (PEGASUS‐PCI^17^ and ATLANTIS‐ACS^5^). Both studies were prospective observational trials performed at the Medical University of Vienna and Medical University of Graz and approved by Ethics Committees in accordance with the Declaration of Helsinki. Patients were included into the studies between August 2006 and June 2015 in Vienna and Graz. Only patients who underwent an urgent percutaneous coronary intervention (PCI) due to ACS and who were treated with drug‐eluting stents (DES) were selected from the two cohorts: 571 patients from the ATLANTIS‐ACS study and 137 patients from PEGASUS‐PCI study. In short, consecutive patients with a written informed consent obtained before the study entry, treatment with P2Y12 inhibitors and age >18 years were included. The only exclusion criterion in both studies was participation in other interventional trials. Patients received a loading dose of a P2Y12 inhibitor before PCI followed by a maintenance dose for a planned duration of 12 months.^17^, ^18^ In both study centres, the used type of P2Y12 inhibitor was at the discretion of the interventional cardiologist in charge, which was in accordance with the current guidelines at time point of intervention and intern standard operating procedures (SOP) for antiplatelet therapy in patients after ACS. All patients received unfractionated heparin for the ACS diagnosis and also during the PCI. In the PEGASUS‐PCI trial blood was obtained and analysed immediately post‐PCI. In the ATLANTIS‐ACS study, blood was collected at least one day after loading during the treatment with maintenance doses of prasugrel and ticagrelor. Clinical follow‐up information was obtained by contacting all patients by phone and/or mail every three months during the first year of follow‐up in both studies. In addition, information concerning the long‐term mortality and cause of death was obtained from the national death registry. Patients were followed‐up for a median of 2059 days (ie 5.6 years; interquartile range [IQR] 4.9‐6.5 years; minimum: 360 days; maximum: 7.2 years). Only 5 (0.7%) patients were lost at follow‐up. A total of 71 (10%) patients discontinued their antiplatelet therapy within 12 months due to adverse events. This study uses the STROBE (strengthening the reporting of observational studies in epidemiology) standards and recommendations (Supplementary file). Reporting of the study conforms to broad EQUATOR guidelines.^19^

### 2.2 Study endpoints

Primary efficacy endpoint was long‐term mortality and incidence of MACE within one year after discharge compared between the clopidogrel, ticagrelor and prasugrel group. MACE was defined as nonfatal myocardial infarction, nonfatal stroke or cardiovascular death. Secondary endpoint was the distribution of adenosine diphosphate (ADP)‐ and arachidonic acid (AA)‐induced platelet aggregation with regard to the cause of hospitalization and status of diabetes. Furthermore, we examined predictors of MACE and long‐term mortality. Additionally, we investigated the incidence of stent thrombosis within one year after PCI. Stent thrombosis (definite or probable) was defined as an angiographic or pathologic confirmed thrombosis which led to the occurrence of ACS.^20^ This definition is in accordance with the Academic Research Consortium criteria.^21^ Thrombolysis in myocardial infarction (TIMI) major, minor and minimal bleeding was considered a safety endpoint.^22^

### 2.3 Impedance aggregometry

To determine whole blood aggregation, multiple electrode aggregometry (MEA) was used on a new generation impedance aggregometer (Multiplate Analyzer, Roche Munich, Germany). Electrical impedance change due to platelet adhesion and aggregation was detected by two independent electrode‐set surfaces in the test cuvette as described.^23^, ^24^, ^25^, ^26^ Hirudin was the anticoagulant of choice; ADP and AA were used as agonists. An 1:2 dilution of whole blood anticoagulated with hirudin and 0.9% NaCl was stirred at 37°C for 3 minutes in the test cuvettes. Further, ADP in a concentration of 6.4 µmol/L and AA in a concentration of 0.5 mmol/L were added and the increase in electrical impedance was recorded continuously for 6 minutes. The mean values of the 2 independent determinations are expressed as U (units). According to previous literature, values >46 U were considered as high on treatment platelet reactivity (HTPR).^17^ MEA was performed at the Department of Cardiology at the Medical University of Vienna.

### 2.4 Statistical analysis

Based on an 18% mortality in the clopidogrel group as compared to 8% in the prasugrel/ticagrelor groups, we calculated that with 708 patients (1:4 sampling ratio), our analysis had an 80% power with a two‐sided alpha value of <0.05. Data are expressed as mean, standard deviation (SD), 95% confidence intervals (CI), median, and interquartile range (IQR) as appropriate. Statistical comparisons were performed with the Kruskal‐Wallis test, the Mann‐Whitney *U* test, and the chi‐square test when appropriate. For survival analysis, we used Kaplan‐Meier curves with the Mantel‐Cox regression test. Classification tree analysis with chi‐squared automatic interaction detection (CHAID) was used to detect discriminators for incidence of MACE and long‐term mortality. The classification tree analysis included variables such as sex, age category (≤65, >65 years), comorbidities (hypertension, diabetes, dyslipidaemia, peripheral vascular disease), smoking history, body mass index (BMI) and type of ACS (ST segment elevation myocardial infarction [STEMI] vs non‐ST elevation myocardial infarction [NSTEMI] vs unstable angina). Multivariate Cox regression analysis was used to determine independent variables responsible for clinical outcome. The model included the following variables: use of clopidogrel or ticagrelor or prasugrel, sex, age category (≤65, >65 years), current smoking, hypertension, dyslipidaemia, reason for hospitalization, previous myocardial infarction, previous PCI , concomitant medication (angiotensin converting enzyme [ACE] inhibitors, statins, calcium channel blockers), HTPR phenotype and the time point of inclusion (before or after 2010). Variables were chosen according to significant differences between the subgroups at baseline. All statistical calculations were performed using commercially available statistical software (IBM SPSS Statistics 25, IBM, Armonk/New York, United States of America).

## 3 RESULTS

### 3.1 Patient demographics

Patient demographics, concomitant medication and ACS data are shown in Table 1: this study included 708 patients, 137 patients (19%) were treated with clopidogrel, 260 patients (37%) with ticagrelor, and 311 patients (44%) with prasugrel for a duration of 12 months. There was a higher proportion of men in all three subgroups (overall 78%). The majority of patients had cardiovascular risk factors such as hypertension (66%), dyslipidaemia (57%) and a history of smoking (66%), while family history of coronary artery disease (CAD) has only been reported in 34%. Diabetes mellitus was diagnosed in 23% of the patients. Use of proton pump inhibitors (86%), beta‐blockers (90%), statins (95%) and ACE inhibitors (88%) was high due to the initiated ACS treatment. More patients with unstable angina received clopidogrel (20% vs 4% ticagrelor vs 2% prasugrel; *P* < .001). Ticagrelor was mostly used in patients with NSTEMI (62% vs 40% clopidogrel vs 8% prasugrel; *P* < .001) and prasugrel predominantly in patients diagnosed with STEMI (90% vs 40% clopidogrel vs 34% ticagrelor; *P* < .001). Furthermore, prasugrel‐treated patients were younger than clopidogrel and ticagrelor‐treated patients (57 years vs 63 years vs 63 years; *P* < .001) and more frequently male (84% vs 75% vs 72%; *P* = .002). Hypertension was more often reported in patients treated with clopidogrel compared to patients treated with ticagrelor or prasugrel (79% vs 66% vs 60%; *P* < .001) as well as dyslipidaemia (76% vs 55% vs 50%; *P* < .001), peripheral arterial occlusive disease (10% vs 4% vs 5%; *P* = .026) and a prior myocardial infarction (27% vs 17% vs 18%; *P* = .049) or a prior PCI (44% vs 22% vs 23%; *P* < .001). Additionally, HTPR phenotype occurred more frequently in the clopidogrel group as compared to the ticagrelor or prasugrel group (44% vs 3% vs 4%; *P* < .001). On the other hand, patients in the prasugrel group were more likely to have a history of smoking (74% vs 63% vs 58%; *P* < .001). Statins we more frequently administered in the prasugrel and ticagrelor groups (97% vs 95% vs 88%; *P* = .001), as well as ACE inhibitors (93% vs 90% vs 77%; *P* < .001). Calcium channel blockers were used more often in the clopidogrel and ticagrelor group than in the prasugrel group (13% vs 12% vs 6%; *P* = .009).

**TABLE 1:** Patient demographics

Patient demographics	Overall N = 708	Clopidogrel N = 137 (19)	Ticagrelor N = 260 (37)	Prasugrel N = 311 (44)	*P*‐value
Age (years) mean ± SD	60 ± 12.4	63 ± 12.8	63 ± 12.6	57 ± 11.2	<.001
Sex (male) n (%)	553 (78)	103 (75)	188 (72)	262 (84)	.002
Risk factors/past medical history n (%)					
Body mass index (BMI) mean ± SD	28 ± 4.5	28 ± 3.9	28 ± 4.5	28,2 ± 4.8	.622
Hypertension	463 (66)	107 (79)	171 (66)	185 (60)	<.001
Dyslipidaemia	400 (57)	103 (76)	143 (55)	154 (50)	<.001
Diabetes mellitus	164 (23)	35 (26)	61 (24)	68 (22)	.676
Peripheral arterial occlusive disease	40 (6)	14 (10)	10 (4)	16 (5)	.026
Cerebrovascular disease	22 (3)	3 (2)	11 (4)	8 (3)	.422
Smoking (past)	464 (66)	85 (63)	149 (58)	230 (74)	<.001
Smoking (present)	294 (46)	54 (40)	83 (36)	157 (58)	<.001
Family history of CAD	237 (34)	33 (24)	83 (32)	121 (39)	.009
Prior myocardial infarction	136 (19)	36 (27)	45 (17)	55 (18)	.049
Prior PCI	188 (27)	59 (44)	57 (22)	72 (23)	<.001
HTPR phenotype	80 (12)	60 (44)	7 (3)	13 (4)	<.001
Laboratory data (mean ± SD)					
Platelets (×10^9^/L)	209.5 ± 92.9	223.9 ± 74.5	214.8 ± 96.8	199 ± 95.8	.145
Concomitant medication n (%)					
Aspirin	708 (100)	137 (100)	260 (100)	311 (100)	
Proton pump inhibitors (PPI)	597 (86)	115 (85)	226 (88)	256 (84)	.491
ß‐blockers	631 (90)	124 (91)	228 (88)	279 (92)	.371
Statins	660 (95)	120 (88)	246 (95)	294 (97)	.001
Angiotensin converting enzyme (ACE) inhibitors	611 (88)	97 (71)	232 (90)	282 (93)	<.001
Calcium channel‐blockers	66 (10)	17 (13)	32 (12)	17 (6)	.009
Antidiabetic drugs	120 (17)	30 (22)	46 (18)	44 (15)	.141
ACS data					
Unstable angina	44 (6)	27 (20)	11 (4)	6 (2)	<.001
NSTEMI	241 (34)	55 (40)	160 (62)	26 (8)	<.001
STEMI	423 (60)	55 (40)	89 (34)	279 (90)	<.001
Number of stents per patient	1.6 ± 1.1	1.8 ± 1.2	1.6 ± 1.2	1.5 ± 0.9	.130
Total stent length	32.7 ± 22.2	34.8 ± 25	33.8 ± 23.9	30.9 ± 19.2	.878

Note

Data are reported as Mean ± standard deviation (SD), n (number of patients) or percentages; CAD, coronary artery disease; PCI, percutaneous coronary intervention; PCI, percutaneous coronary intervention; STEMI, ST elevation myocardial infarction; NSTEMI, non‐ST elevation myocardial infarction; HTPR, high on treatment platelet reactivity.

### 3.2 Distribution of ADP‐induced platelet aggregation with regard to the administered drug and event type

**FIGURE 1:**
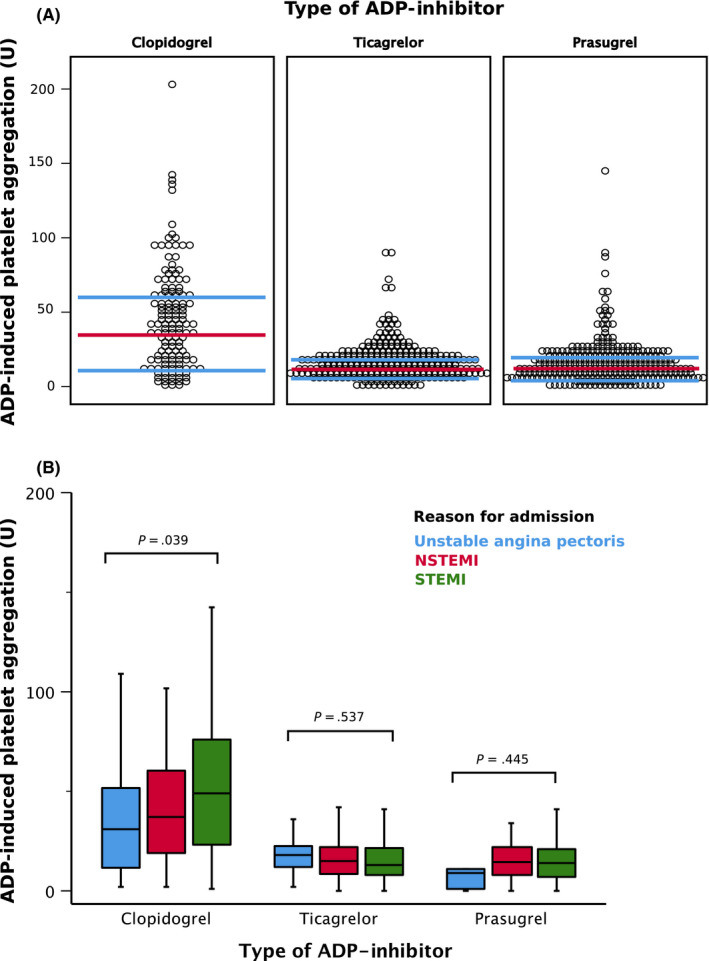
Adenosine diphosphate (ADP)‐induced platelet aggregation assessed by multiple electrode aggregometry in relation to (A) the type of ADP inhibitor and (B) diagnosis at hospitalization

Median level of ADP‐induced platelet aggregation was 67% higher in the clopidogrel group (median 42U; IQR 18‐63U; Figure 1A) as compared to the ticagrelor (14U; IQR 8‐22U; *P* < .001) or prasugrel groups (14U; IQR 7‐22U; *P* < .001). The level of ADP‐induced platelet aggregation varied between patients presenting with unstable angina, NSTEMI or STEMI in clopidogrel treated patients: the clopidogrel effect on counteracting the binding of ADP on the P2Y12 receptor was less effective with increasing severity of ACS (unstable angina: 31U; IQR 12‐54U; NSTEMI: 37U; IQR 19‐60U; STEMI: 49U; IQR 23‐76U; *P* = .039). Relatively expressed, clopidogrel acted stronger by 37% in unstable angina or 25% in NSTEMI than in STEMI. There was no difference between the three ACS groups in patients treated with ticagrelor (*P* = .537) or prasugrel (*P* = .445; Figure 1B).

### 3.3 Distribution of ADP‐ and AA‐induced platelet aggregation values with regard to diabetes mellitus

**FIGURE 2:**
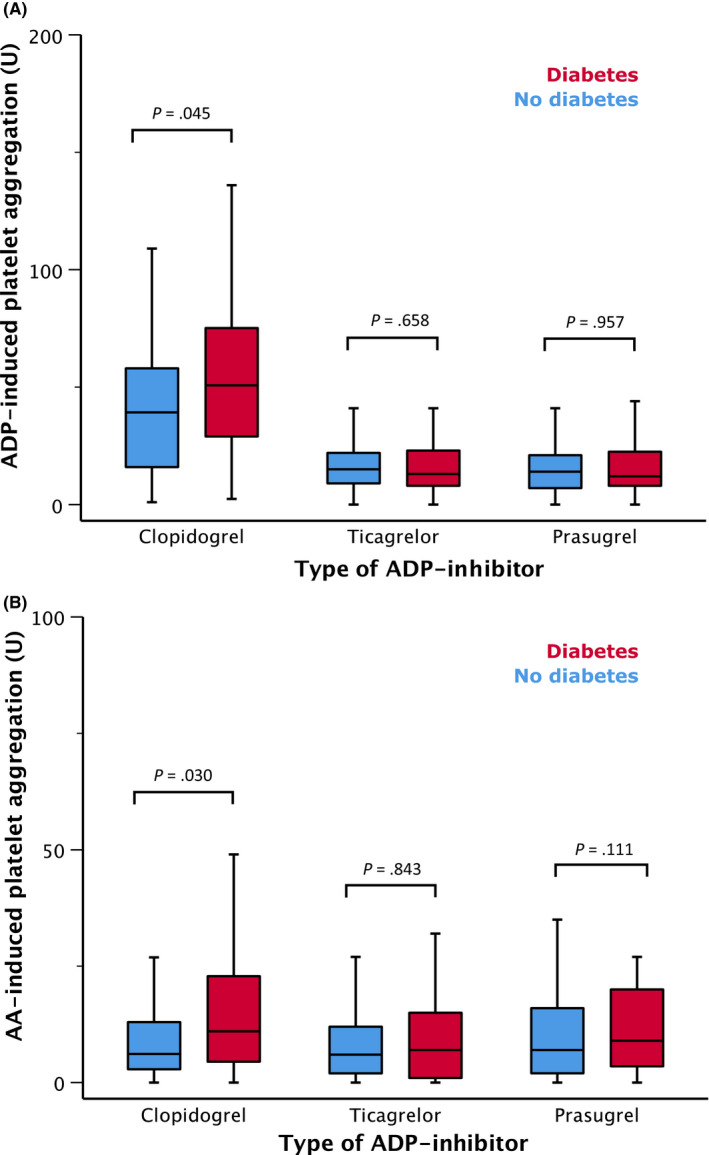
(A) ADP‐induced and (B) arachidonic acid (AA)‐induced platelet aggregation in regard to type of ADP inhibitor and status of diabetes

Median levels of ADP‐induced platelet aggregation were 24% higher in clopidogrel‐treated patients with diabetes (51U; IQR 29‐75U) vs without diabetes (39U; IQR 16‐58U; *P* = .045; Figure 2A). There was no difference in the ADP‐induced platelet reactivity between patients with diabetes and patients without diabetes under treatment with ticagrelor (15U; IQR 9‐22U vs 13U; IQR 8‐23U; *P* = .658) or prasugrel (14U; IQR 7‐21U vs 12U; IQR 8‐22U; *P* = .957; Figure 2A).

AA‐induced aggregation was higher by 46% in patients with diabetes vs patients without diabetes treated with clopidogrel (6U; IQR 3‐13U vs 11U; IQR 5‐23U; *P* = .03). There was no difference in the AA‐induced platelet reactivity between patients with diabetes and patients without diabetes under treatment with ticagrelor (6U; IQR 2‐12U vs 5U; IQR 9‐22U; *P* = .843; Figure 2B) or prasugrel (7U; IQR 2‐16U vs 9U; IQR 4‐20U; *P* = .111; Figure 2B).

### 3.4 Predictors of major adverse cardiac events

**FIGURE 3:**
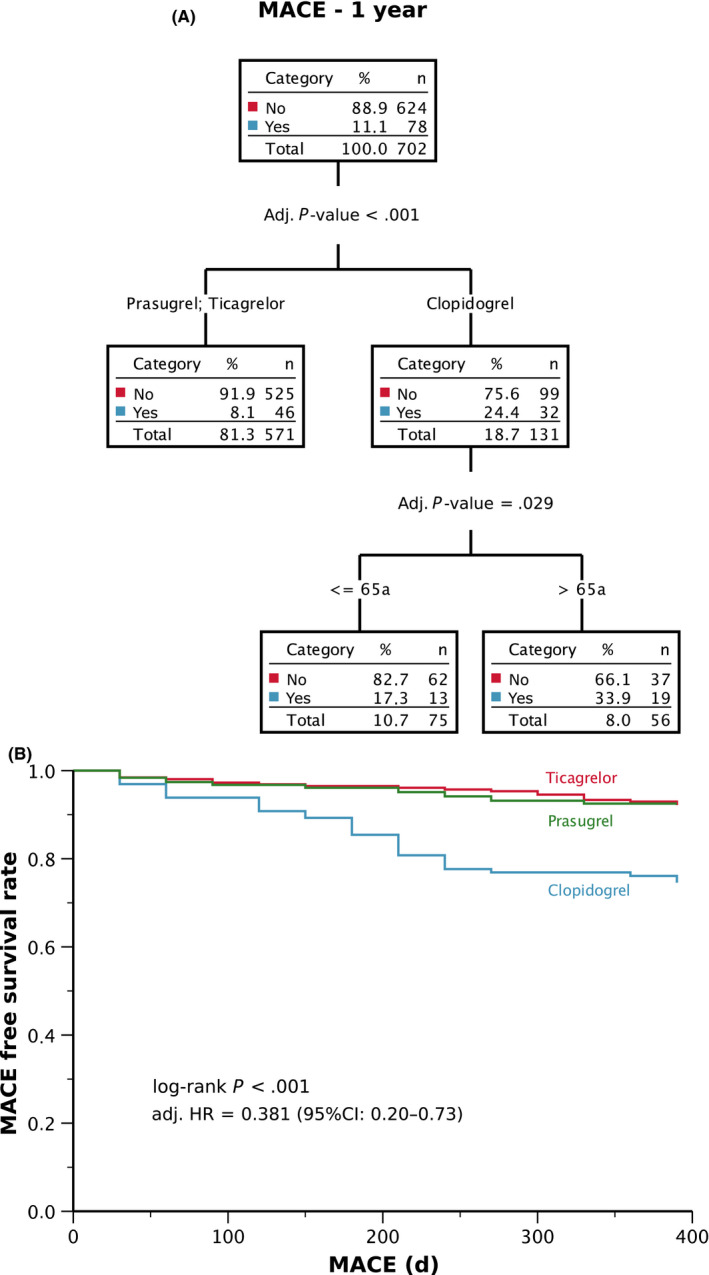
(A) Chi‐squared automatic interaction detection (CHAID) for predictors of MACE; (B) Kaplan‐Meier survival analysis for MACE

CHAID was used to find predictors for MACE during the follow‐up time of one year. Overall 11.1% of the study cohort had a MACE during the period of one year. Age (≤/>65 years) and the type of P2Y12 inhibitor (clopidogrel, ticagrelor or prasugrel) emerged as significant discriminators of MACE (Figure 3A). The most powerful predictor for MACE represented the type of used P2Y12 inhibitor: 24% of the patients treated with clopidogrel experienced a MACE, while such events occurred in only 8% of patients who were treated with ticagrelor or prasugrel (*P* < .001; Figure 3A). Age influenced solely the outcome of patients in the clopidogrel group with 17.3% of patients younger than or equal to 65 years reported MACE vs 33.9% of patients older than 65 years of age (*P* = .029).

The time to event analysis has confirmed that the composite endpoint of MACE occurred significantly less often in patients treated with ticagrelor or prasugrel than in patients administered to clopidogrel (8% vs 8% vs 24%; *P* < .001; Figure 3B) during the 12 months follow‐up. Although MACE events occurred more often in patients with HTPR phenotype as compared to no HTPR (22% vs 10%; *P* = .001), this association lost its significance after adjustment. The multivariate Cox regression analysis confirmed that the risk to develop MACE was 62% lower in the ticagrelor/prasugrel group as compared to the clopidogrel group (adjusted hazard ratio [HR] = 0.38; 95%CI: 0.20‐0.73; *P* = .004; Figure 3B).

### 3.5 Predictors for long‐term mortality

**FIGURE 4:**
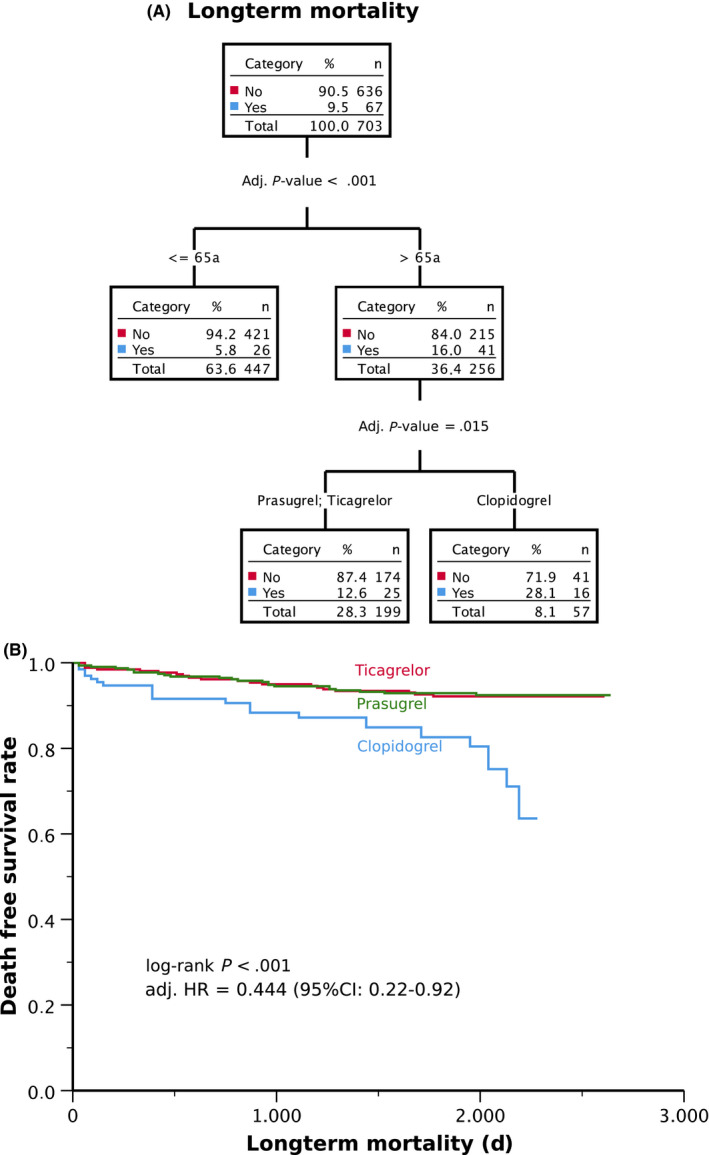
(A) Chi‐squared automatic interaction detection (CHAID) for predictors of long‐term mortality; (B) Kaplan‐Meier survival analysis for long‐term mortality

The type of P2Y12 inhibitor and age were the only variables influencing the outcome. The most powerful discriminator of mortality was age. During the median follow‐up time of 5.6 years, 9.5% of patients died: 5.8% of those ≤65years of age and 16% of those >65years of age (*P* < .001; Figure 4A). Those older than 65 years of age had a 2.2‐fold lower risk to die if treated with ticagrelor or prasugrel vs clopidogrel (mortality: 12.6% vs 28.1%; *P* = .015). There was no difference in the risk of mortality between prasugrel and ticagrelor.

Also, in the overall study population, long‐term mortality was lower in the ticagrelor/prasugrel group compared to the clopidogrel group (8% vs 7% vs 18%; *P* = .001; respectively; Figure 4B). Patients with HTPR had higher long‐term mortality as compared to patients without HTPR (22% vs 8%; *P* < .001), which however lost significance in the multivariable analysis adjusted for P2Y12 inhibitor type. The multivariate Cox regression showed a 56% risk reduction of death in the ticagrelor/prasugrel group vs the clopidogrel group (adjusted HR = 0.44; 95%CI: 0.22‐0.92; *P* = .028).

### 3.6 Incidence of stent thrombosis

Overall stent thrombosis occurred in 8 patients (2%) within one year after PCI as displayed in Table 2. The highest incidence of stent thrombosis was found in the clopidogrel group as compared to the ticagrelor or prasugrel groups (4% vs 1.4% vs 0.6%; *P* < .001; respectively). Cox regression analysis showed that the type of ADP inhibitor missed the statistical significance as an independent predictor for stent thrombosis (crude HR = 0.25; 95%CI: 0.06‐1.05; *P* = .057; adjusted HR = 0.32; 95%CI: 0.04‐2.29; *P* = .255).

**TABLE 2:** Clinical outcome data

Event n (%)	Overall	Clopidogrel	Ticagrelor	Prasugrel	*P*‐value
TIMI Major bleeding (1 year)	5 (0.7)	1 (0.8)	2 (0.8)	2 (0.6)	.977
MACE (1 year)	78 (11)	32 (24)	21 (8)	25 (8)	<.001
Stent thrombosis (1 year)	8 (2)	5 (4)	2 (1.4)	1 (0.6)	<.001
Long‐term mortality	67 (10)	24 (18)	20 (8)	23 (7)	.001

Note

Data are reported as n (number of patients) and percentages; TIMI, thrombolysis in myocardial infarction; MACE, major adverse cardiac events.

### 3.7 Safety of treatment: bleeding complications

Overall, only 5 TIMI major bleeding events occurred in the study cohort (0.7%) during 12‐months follow‐up with no statistically significant difference between the groups (Table 2).

## 4 DISCUSSION

The central findings of this study investigating differences in clinical outcome and pharmacodynamic effects between clopidogrel, ticagrelor, and prasugrel are as following:

1. The administered type of P2Y12 inhibitor is the main predictor of MACE and it is also a predictor of long‐term mortality.
2. The clopidogrel effect decreases with increasing severity of ACS.
3. Patients with diabetes under clopidogrel treatment displayed higher ADP‐ and AA‐induced platelet aggregation when compared to those without diabetes.

This study demonstrated that the administered type of ADP inhibitor is the crucial discriminator of MACE during a follow‐up period of one year. Patients treated with ticagrelor or prasugrel had statistically significant better outcomes compared to those treated with clopidogrel. Superiority of potent P2Y12 inhibitors compared to clopidogrel in patients with ACS is a well‐described finding with respect to reduce the incidence of myocardial infarction or cardiovascular death after an ACS.^16^ Our study also confirms findings of large observational studies, which compared clinical outcomes in ACS patients, who underwent PCI and were treated with clopidogrel, ticagrelor or prasugrel. Ticagrelor and prasugrel had lower incidence of the composite endpoint of cardiac death, nonfatal myocardial infarction or stroke.^27^ Our findings therefore support the current ESC guidelines, which recommend clopidogrel only for stable CAD, whereas ticagrelor or prasugrel is the preferred therapy in patients with ACS.^13^, ^14^, ^15^ Interestingly, the recent ISAR‐REACT 5 trial showed inferiority of ticagrelor strategy compared to prasugrel strategy for the primary composite endpoint of myocardial infarction, stroke or death (HR = 1.36; 95% CI: 1.09‐1.70; *P* = .006).^28^ In contrast to the ISAR‐REACT 5 trial (which was a strategy comparison), all patients in our study received the study drug before PCI and were discharged on a study drug. Therefore, our study was not a strategy comparison, but an observation on the effects of clopidogrel, ticagrelor and prasugrel on clinical outcome.

Importantly, another significant predictor of MACE in our study was age >65 years in patients treated with clopidogrel but not ticagrelor or prasugrel. This translates into an assumption that especially older patients at a higher risk of MACE may benefit the most if treated with prasugrel or ticagrelor. Indeed, age has been shown in several studies as a predictor of HTPR, which in turn correlates with worse clinical outcome.^29^, ^30^ Furthermore, it has been shown that elderly patients (≥75 years) diagnosed with STEMI had lower risk of MACE within one year when treated with ticagrelor instead of clopidogrel (HR = 0.69; 95% CI: 0.49‐0.97; *P* = .03).^31^ In the past few years, also the platelet‐to‐lymphocyte ratio (PLR) has been demonstrated to be a promising predictor for MACE in ACS patients.^32^, ^33^ Studies showed that higher values of PLR were associated with an increased occurrence of cardiovascular events in patients after ACS.^34^, ^35^

Of interest, the main predictor of long‐term mortality was age, which is a constant finding.^36^ The second predictor of mortality was the type of the used P2Y12 inhibitor underlining its importance even with regard to long‐term outcomes. The PLATO trial and the TRITON‐TIMI 38 trial had a median follow‐up time of 9 and 15 months, respectively.^10^, ^11^ In our study, median follow‐up period was 5.6 years. Therefore, the observation of long‐term mortality rates between clopidogrel, ticagrelor and prasugrel is a novel aspect of our study.

Another interesting finding is the homogenous antiplatelet effect of ticagrelor and prasugrel as assessed with platelet aggregation testing, independent from the type of ACS or the diabetes status. In contrast, patients administered clopidogrel showed statistically significant variation in ADP‐induced platelet aggregation according to the ACS type. With increasing severity of the disease (STEMI > NSTEMI > unstable angina), the effect of clopidogrel decreased, which was demonstrated as elevated platelet reactivity in the MEA. These findings may be explained by HTPR on clopidogrel due to insufficient inhibition, occurring in about 20%‐30% of patients.^37^, ^38^, ^39^, ^40^, ^41^ Ticagrelor and prasugrel are superior to clopidogrel in reducing HTPR.^42^, ^43^, ^44^ HTPR on clopidogrel due to increasing severity of CAD would provide a possible explanation for the gradual rise in platelet aggregation levels according to the diagnosis at hospitalization. However, it has been suggested that HTPR also occurs in patients treated with prasugrel or ticagrelor, especially those after cardiac arrest, therapeutic hypothermia or concomitant use of morphine.^45^, ^46^, ^47^ In our investigation, the pharmacodynamic effects between prasugrel and ticagrelor were similar. This finding contrasts with some previous studies, where ticagrelor produced more potent platelet inhibition and was associated with lower HTPR rates than prasugrel.^41^, ^42^ It has been suggested that HTPR on clopidogrel treatment might be a predictor of HTPR on prasugrel. The SWAP‐2 study showed that switching from ticagrelor to prasugrel therapy was associated with higher levels of platelet reactivity as compared to continuous ticagrelor treatment.^43^ Another randomized pharmacodynamic trial reported that ACS patients treated with ticagrelor had lower platelet reactivity as compared to patients administered to prasugrel.^44^ Consequently, it seems that several variables might have an influence on the results, such as previous treatment with clopidogrel, loading sequence, time point of investigation, strategy of switching or patient population.

Another important aspect of our study was the higher level of platelet reactivity in patients with diabetes mellitus. We found that patients with diabetes receiving clopidogrel had significantly higher platelet reactivity compared to those treated with ticagrelor or prasugrel. This was mirrored in elevated levels of ADP‐induced platelet aggregation, as well as in AA‐induced platelet aggregation in patients with diabetes under clopidogrel, which goes in line with previous findings.^48^, ^49^ A possible explanation for that phenomenon could be higher levels of soluble P‐selectin in patients diagnosed with CAD and diabetes leading to increased platelet reactivity.^50^ Another study showed a reduced amount of circulating active clopidogrel metabolites in patients with diabetes due to its insufficient transformation to an active drug.^51^ Although prasugrel has to be metabolized as well, it provides a higher bioactivation, which leads to improved platelet inhibition and reduced variability in patients with diabetes.^52^ Furthermore, higher levels of inflammatory markers in patients with diabetes might be associated with decreased inhibitory response to clopidogrel, as well as to aspirin.^53^ Additionally, platelets in patients with diabetes are known to be hyperactive in terms of adhesion and activation as well as aggregation.^54^, ^55^ Ticagrelor is an active drug with adenosine‐mediated anti‐inflammatory effects, which might provide some explanation for the improved outcomes in patients with diabetes.^56^ In our study, pharmacodynamic effects between ticagrelor and prasugrel were similar in patients with diabetes during the maintenance phase of treatment, which is in line with the recent RENAMI substudy, where ticagrelor and prasugrel were compared in patients with diabetes and ACS according to clinical outcome and seem to benefit equally regarding MACE.^57^ However, this finding is in contrast to one previous study, where Laine at al. showed that loading doses (LD) of ticagrelor are superior to LD of prasugrel in reducing platelet reactivity in patients with diabetes.^58^ Interestingly, patients with diabetes treated with clopidogrel in our study had significantly higher levels of AA‐induced platelet aggregation, which might reflect a dual non‐responsiveness to clopidogrel and aspirin treatment.^59^, ^60^ Recently, it has been shown in the THEMIS trial, that ticagrelor plus aspirin were superior in preventing ischemic cardiovascular events in patients with diabetes and stable CAD and PCI as compared to placebo plus aspirin ^61^ at a higher risk of bleeding events. Therefore, the issue of the net clinical benefit in this vulnerable patient population remains a matter of debate.

## 5 LIMITATIONS

There are two main limitations of our study. First, the outcome might be biased by an observational design. Second, clopidogrel was mainly administered before 2010 in our study. Although we used adjustment methods (also for the year of inclusion) in the multivariate Cox regression analysis, bias cannot be excluded. Additionally, we found a high incidence of 1‐year MACE in the clopidogrel group, which might be due to the unselected patient population included into our study. Another minor limitation is that the blood sampling was not exactly at the same time point for each patient. Nevertheless, the level of platelet aggregation shown in our study is in the same range as previously published in many reports, also in the pharmacodynamic regulatory studies on those three drugs. Based on that assumption, this limitation has most probably a very low impact on the results.

## 6 CONCLUSION

Long‐term outcome data confirm superiority of ticagrelor and prasugrel over clopidogrel with respect to mortality and MACE, which is due to their potent and homogenous effect independent from clinical characteristics.

## AUTHORS' CONTRIBUTIONS

Gloria M. Gager analysed and interpreted the data, drafted the manuscript and finally approved the manuscript submitted. Bernd Jilma involved in study conception and design, revised it critically for important intellectual content and finally approved the manuscript submitted. Max‐Paul Winter involved in patients' follow‐up, revised it critically for important intellectual content and finally approved the manuscript submitted. Christian Hengstenberg revised it critically for important intellectual content and finally approved the manuscript submitted. Irene M. Lang involved in patient inclusion and blood sampling, revised it critically for important intellectual content and finally approved the manuscript submitted. Aurel Toma revised it critically for important intellectual content and finally approved the manuscript submitted. Florian Prüller, Markus Wallner and Ewald Kolesnik involved in patient inclusion and follow‐up, revised it critically for important intellectual content and finally approved the manuscript submitted. Dirk von Lewinski involved in conception, design, patient inclusion, follow‐up and platelet aggregation assessment; revised it critically for important intellectual content; and finally approved the manuscript submitted. Jolanta M. Siller‐Matula involved in conception, design, analysis and interpretation of data, patient inclusion, blood sampling, platelet aggregation assessment and manuscript drafting; revised it critically for important intellectual content; and finally approved the manuscript submitted.

## Supporting information

Supplementary Material
